# Effective continuous acetone–butanol–ethanol production with full utilization of cassava by immobilized symbiotic TSH06

**DOI:** 10.1186/s13068-019-1561-1

**Published:** 2019-09-16

**Authors:** Zhangnan Lin, Hongjuan Liu, Jing Wu, Petra Patakova, Barbora Branska, Jianan Zhang

**Affiliations:** 1 Institute of Nuclear and New Energy Technology, Tsinghua University, Beijing, 100084 China; 2 Department of Biotechnology, University of Chemistry and Technology Prague, Technicka 5, 16628 Prague 6, Czech Republic

**Keywords:** ABE fermentation, Symbiotic TSH06, Cassava utilization, Cell immobilization, Continuous fermentation

## Abstract

**Background:**

Butanol production by fermentation has recently attracted increasingly more attention because of its mild reaction conditions and environmentally friendly properties. However, traditional feedstocks, such as corn, are food supplies for human beings and are expensive and not suitable for butanol production at a large scale. In this study, acetone, butanol, and ethanol (ABE) fermentation with non-pretreated cassava using a symbiotic TSH06 was investigated.

**Results:**

In batch fermentation, the butanol concentration of 11.6 g/L was obtained with a productivity of 0.16 g/L/h, which was similar to that obtained from glucose system. A full utilization system of cassava was constructed to improve the fermentation performance, cassava flour was used as the substrate and cassava peel residue was used as the immobilization carrier. ABE fermentation with immobilized cells resulted in total ABE and butanol concentrations of 20 g/L and 13.3 g/L, which were 13.6% and 14.7% higher, respectively, than those of free cells. To further improve the solvent productivity, continuous fermentation was conducted with immobilized cells. In single-stage continuous fermentation, the concentrations of total ABE and butanol reached 9.3 g/L and 6.3 g/L with ABE and butanol productivities of 1.86 g/L/h and 1.26 g/L/h, respectively. In addition, both of the high product concentration and high solvent productivity were achieved in a three-stage continuous fermentation. The ABE productivity and concentration was 1.12 g/L/h and 16.8 g/L, respectively.

**Conclusions:**

The results indicate that TSH06 could produce solvents from cassava effectively. This study shows that ABE fermentation with cassava as a substrate could be an efficient and economical method of butanol production.

## Introduction

In recent years, butanol production by fermentation has attracted much attention due to the concerns of environmental pollution and the shortage of fossil fuels [1]. However, the major obstacles for the industrialization of acetone, butanol, and ethanol (ABE) fermentation were the high cost of the raw materials and the shortage of food. Thus, ABE fermentation with low-cost and non-grain crops is one of the economical and promising methods for butanol production [2].

Cassava, as a starch-rich plant, can grow in poor soils and has a strong adaptability. Africa, the Americas, and Asia are the top three regions for cassava production with a total production of over 276 million tons. Furthermore, cassava is a comparatively cheap and non-grain crop in countries such as Thailand, China and Vietnam. Therefore, it is a promising substrate for butanol production due to both geographical and economic considerations [3]. Cassava peel is a byproduct of processing the roots for starch and cassava flour which constitute 11% of the root and cassava peel is a kind of lignocellulosic material which consists of three main components, namely, cellulose, hemicellulose, and lignin [4]. Many studies have focused on the utilization of cassava for ABE fermentation. Li et al. investigated the ABE fermentation with gelatinized cassava flour using a mutant strain *Clostridium acetobutylicum* ART18, 15.8 g/L of butanol was produced with a butanol yield of 0.31 g/g [2]. Thang et al. studied the ABE fermentation characteristics of *Clostridium saccharoperbutylacetonicum* N1-4 with gelatinized cassava flour, which resulted in a butanol concentration of 16.9 g/L and a total ABE concentration of 21.0 g/L [3]. The butanol fermentation with enzymatically hydrolyzed cassava flour as a substrate by *Clostridium beijerinckii* BA101 was studied by Lépiz-Aguilar et al. and 25.71 g/L of butanol was achieved [5]. However, the cassava substrate used in the previous studies was hydrolyzed cassava or gelatinized cassava, and limited research has focused on continuous ABE fermentation by immobilized cells (cassava peel was used as the immobilization carrier) with non-pretreated cassava.

ABE fermentation with cassava is usually conducted in a two-step process, which includes pretreatment by saccharification or gelatinization and fermentation. The elimination of the pretreatment step is helpful for simplifying the whole process and reducing the cost. Moreover, continuous ABE fermentation could significantly overcome the product inhibition of ABE fermentation and thus improve the productivity of ABE [6–9]. In this study, ABE production from cassava was investigated using a symbiotic TSH06. The symbiotic TSH06 isolated in our previous study included *Clostridium acetobutylicum* (CGMCC 8071) and *Bacillus cereus* (CGMCC 8072) and TSH06 is capable of producing butanol under non-strict anaerobic conditions [10, 11]. In addition to superior properties in the tolerance of oxygen, this symbiosis possesses an amylolytic activity to hydrolyze starch. With corn flour medium, 8.96 g/L butanol was obtained and total ABE concentration reached 12.35 g/L [11]. In this study, ABE fermentation with non-pretreated cassava flour was explored using TSH06. All the fermentations were conducted under non-strict anaerobic conditions. To make the butanol production more efficient, single-stage and three-stage continuous ABE fermentations were studied with cell immobilization. Cassava flour was used as a substrate and cassava peel residue was used as the carrier for full-cassava utilization. This work provided the feasibility of ABE fermentation with non-pretreated cassava flour and was helpful for promoting the industrialization development of butanol production from cassava.

## Materials and methods

### Strains

The symbiotic TSH06 used in the current work included *Clostridium acetobutylicum* (CGMCC 8071) and *Bacillus cereus* (CGMCC 8072), which was isolated in previous study [11].

### Mediums

The corn medium (containing 60 g/L corn meal) was heated and boiled for 40 min. Then, the corn medium was autoclaved at 121 °C for 20 min. The corn medium was used for the activation of TSH06.

The P2 medium contained: glucose 65 g/L, yeast extract 1 g/L, K_2_HPO_4_ 0.5 g/L, KH_2_PO_4_ 0.5 g/L, ammonium acetate 2.2 g/L and a mineral and vitamin solution. The mineral and vitamin solution contained: FeSO_4_·7H_2_O 1 g/L, NaCl 1 g/L, MnSO_4_·H_2_O 1 g/L, MgSO_4_·7H_2_O 20 g/L, para-amino-benzoic acid 0.1 g/L, biotin 0.001 g/L and thiamin 0.1 g/L. The mineral and vitamin solution was sterilized by filtration (0.22-μm membrane filters) and then the solution was added to seed medium or fermentation medium at a ratio of 1%. The P2 medium was used for seed culture and fermentation culture.

The cassava medium contained: cassava flour 70 g/L, yeast extract 1 g/L, K_2_HPO_4_ 0.5 g/L, KH_2_PO_4_ 0.5 g/L, ammonium acetate 2.2 g/L and the mineral and vitamin solutions. The composition of the vitamin and mineral solutions was the same as the P2 medium. Cassava medium was used for fermentation culture. The feeding medium used in continuous fermentation was the same as the cassava medium.

### Cassava flour pretreatment

Cassava was purchased from Guangdong Province (GUOXIANHUI), China. The cassava was peeled and chopped into small pieces (1 cm) and then dried at 60 °C to a constant weight and ground into powder. The cassava powder was then pretreated using the following three methods.

#### Enzymatic hydrolysis

NaOH (2 mol/L) was used for adjusting the pH and the pH of the cassava flour suspension was adjusted to 6.5. CaCl_2_ was added the solution at a ratio of 4:100,000. Then, α-amylase (2100 U/g, Aladdin, Tianjin) was added to the cassava flour suspension at 8 U/g-cassava, the sample was stirred and liquefied at 95 °C for 2 h. Then the sample was cooled to 65 °C and the pH was adjusted to 4.5 with HCl (2 mol/L). Subsequently, β-glucoamylase (100,000 U/g, Aladdin, Tianjin) was added at 120 U/g-cassava and the sample was saccharified at 65 °C for 4 h. Finally, the hydrolysate was autoclaved at 115 °C for 15 min.

#### Gelatinization

The cassava flour suspension was heated at 95 °C for 1 h with stirring. Then, the gelatinized sample was autoclaved at 115 °C for 15 min. The pH was not controlled during the process.

#### Non-pretreatment

The cassava flour suspension was heated to 95 °C with stirring until the mixture became a paste (approximately 10 min). Then, the sample was autoclaved at 115 °C for 15 min.

### Culture conditions

#### Bacteria activation

The cell suspension in a glycerol tube was added to a corn medium and the bacteria activation was conducted at 37 °C for 20–24 h.

#### Seed culture

The active bacteria were added to the seed medium. The seed culture was conducted at 30 °C for 18–24 h until the OD600 reached 2.

#### Batch fermentation

Batch fermentations were conducted in 1-L reactors containing 500-mL fermentation medium at 37 °C without stirring and pH control. The inoculation size for fermentation medium was 7%.

#### Continuous fermentation

The culture conditions of continuous fermentation were the same as batch fermentation. When the concentration of butanol reached 8 g/L, the continuous fermentation mode was started. The feeding medium was pumped into the reactor with a peristaltic pump (LONGER BT100-2J) at a feeding rate of 100 mL/h (0.2/h of dilution rate). The same volume of fermentation broth was removed from the reactor to maintain a constant working volume. For three-stage continuous fermentation, when the concentration of butanol in the first-stage reactor increased to 8 g/L (approximately 36 h), the three reactors were connected for continuous fermentation and the feeding medium was continuously fed into the first-stage reactor at a feeding rate of 100 mL/h. At the same time, the broth in each reactor was pumped to the next-stage reactor at a same flow rate, the broth in third-stage reactor was pumped to a storage tank (Additional file 1: Figure S1). The fermentation was conducted without pH control.

### Cell immobilization

The cassava peel residues (20 mesh size) were pretreated with 2 wt% sulfuric acid (the solid/liquid ratio was 1:5) at 130 °C for 60 min. The treated residues were washed with water until neutral and then dried at 60 °C to a constant weight. The dried cassava peel residues were added to the fermentation medium at a ratio of 3.3% (w/w). The cells were then immobilized onto cassava peel residues by adsorption.

### Scanning electron microscope

The immobilized carriers were fixed in 2.5% glutaraldehyde for 4 h. Then, the cassava peel residues were washed 3 times with 0.1 M PBS (pH 7.3–7.4). The samples were dehydrated successively using 50%, 70%, 90%, 95% and 100% ethanol, respectively. The dehydration time for each step is 30 min. Then, the samples were dried overnight in a freeze dryer. The treated samples were sputter-coated with gold–palladium. Subsequently, the residues were examined and photographed by a scanning electron microscope (FEI Quanta 200, America).

### Analytic methods

The biomass was measured with a spectrophotometer at 600 nm (OD600).

Glucose, mannose, produced solvents and acids were analyzed by high-performance liquid chromatography (HPLC) (LC-20AT, Shimadzu, Japan). The samples were diluted to a certain degree and centrifuged and then the supernatants were obtained. An HPX-87H column (Bio-Rad, USA) and a refractive index detector were used, and the samples were detected at 35 °C. 5 mM sulfuric acid was used as the mobile phase with a flowrate of 0.60 mL/min [12].

The determination of starch concentration: a portion of 1 mL cassava medium was added to 9 mL 2 mol/L HCl and then the samples were hydrolyzed at 100 °C for 45 min. An iodine solution was used to determine the hydrolysis of the starch. Then, the pH was adjusted to neutral with 6 mol/L NaOH. Subsequently, the solutions were transferred to 20-mL volumetric flasks and diluted with deionized water to the proper volume. The samples were detected using a biosensor (SBA-40C Jinan Baisheng, China). One gram of starch could be converted into 1.1 g of glucose [13]. The formula used for calculating the starch concentration was [glucose (g/L)/1.1 = starch (g/L)].

The gas produced by the bacteria was measured by a wet gas meter. The fermenter was connected to a wet gas meter and the total evolved gas amount was measured by the wet gas meter.

## Results

### ABE fermentation with pretreated and non-pretreated cassava

Cassava mediums with and without pretreatment were used for ABE fermentation in this study. The cassava pretreatment methods include enzymatic hydrolysis and gelatinization. The results were compared in Table 1, and the fermentation results of glucose culture were used as control group. For the results of glucose culture, 18.3 g/L of total ABE and 11.7 g/L of butanol were produced. The butanol yield and productivity was 0.22 g/g and 0.16 g/L/h, respectively. As shown in Table 1, after the enzymatic hydrolysis of cassava medium, 60.6 g/L glucose was detected and no starch was observed in the cassava hydrolysate medium. At 72 h, 17.5 g/L of total ABE was achieved with a butanol concentration of 11.6 g/L. The productivities of total solvents and butanol were 0.24 and 0.16 g/L/h, respectively.

**Table 1:** ABE fermentation with different pretreated cassava

Parameters	Cassava			Glucose
	Enzymatic hydrolysis	Gelatinization	Non-pretreatment	
Initial starch (g/L)	0	54.4 ± 1.0	54.2 ± 0.9	0
Initial glucose (g/L)	60.6 ± 1.1	0	0	60.3 ± 1.1
Ultimate starch (g/L)	0	0	0	0
Ultimate glucose (g/L)	6.0 ± 1.2	4.9 ± 0.6	4.8 ± 0.9	6.3 ± 1.4
Butanol (g/L)	11.6 ± 0.4	11.4 ± 0.2	11.6 ± 0.3	11.7 ± 0.2
Acetone (g/L)	4.2 ± 0.2	4.2 ± 0.3	4.3 ± 0.1	4.9 ± 0.2
Ethanol (g/L)	1.7 ± 0.1	1.6 ± 0.2	1.6 ± 0.2	1.7 ± 0.1
Total solvents (g/L)	17.5 ± 0.8	17.2 ± 0.7	17.5 ± 0.3	18.3 ± 0.4
Total solvents yield (g/g)	0.35^b^	0.34^b^	0.35^b^	0.33^a^
Butanol yield (g/g)	0.23^d^	0.23^d^	0.23^d^	0.22^c^
Butanol productivity (g/L/h)	0.16	0.16	0.16	0.16
Total solvent productivity (g/L/h)	0.24	0.24	0.24	0.25

^a^Solvent yield on reducing sugar

^b^Solvent yield on cassava starch

^c^Butanol yield on reducing sugar

^d^Butanol yield on cassava starch

When gelatinized cassava or non-pretreated cassava was used as the substrate, the initial cassava flour concentration was 70 g/L, and 54.0 g/L starch was detected in the medium. No starch was detected in the cassava mediums at 96 h, which showed that all starch was hydrolyzed by the TSH06. In addition, both of the two cassava mediums could be utilized by the strain for ABE synthesis and 11.4 g/L and 11.6 g/L of butanol were produced, respectively, with similar butanol productivities of 0.16 g/L/h and butanol yields of 0.23 g/g. The results suggest that TSH06 could grow and produce ABE not only in a medium containing hydrolyzed sugar but also in a medium containing starch. With the cassava flour as the substrate, both the solvent concentrations and productivities were comparable to that of glucose medium.

The fermentation results indicate that TSH06 could efficiently produce solvents from cassava. Since fermentation with non-pretreated cassava medium is advantageous for cost reduction and the simplification of the process, the method of fermentation with non-pretreated cassava medium is the preferred process. In the subsequent study, the non-pretreated cassava medium is used for ABE fermentation.

### Comparison of batch fermentation with free and immobilized cells

**Fig. 1:**
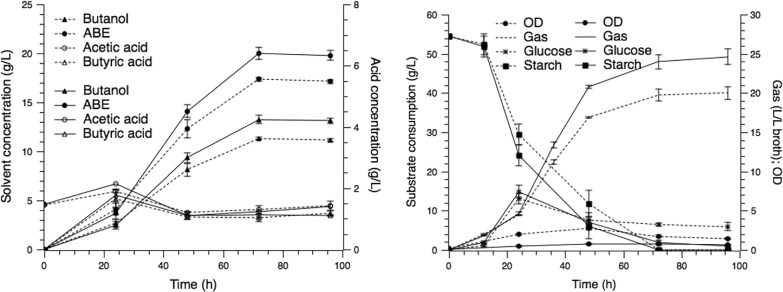
ABE fermentation with free (dashed lines) and immobilized (solid lines) cells

ABE fermentation performance could be improved significantly using the technique of cell immobilization because of the better butanol tolerance and higher cell density [8, 9]. Up to now, a lot of researches have been conducted on ABE fermentation with immobilized cells. Lignocellulosic materials with a porous structure, such as corn stalks, sugar cane bagasse and wood pulp, have been proved to be good choices of carriers [14, 15]. However, limited researches have focused on using cassava as a substrate together with cassava peel residues as immobilized carriers. To evaluate the feasibility and applicability of the full utilization system of cassava, batch fermentations using cassava flour (70 g/L) as a substrate with free cells and immobilized cells were compared and the results are shown in Fig. 1. Starch testing of cassava peel with iodine solution is shown in Additional file 1: Figure S2.

After 72 h of fermentation, 17.6 g/L of total ABE was detected in the free cell system with a yield of 0.35 g/g, and the butanol concentration of 11.6 g/L was obtained. The productivities in the free cell system were 0.16 g/L/h for butanol and 0.25 g/L/h for total solvents. The butanol synthesis in the immobilized cell system and free cell system were similar in the first 24 h, both butanol concentrations were between 2 and 2.5 g/L. After 24 h, a higher rate of butanol synthesis was observed in the immobilized fermentation, which was 27.8% higher than that of free cells. At the end of the fermentation, 13.3 g/L of butanol and 20.0 g/L of total solvents were achieved, which increased by 14.7% and 13.6%. The productivity of butanol reached 0.18 g/L/h, with 25% improvement compared to that of free cells.

Figure 1 shows the kinetic curves of gas production and OD in the immobilized and free cell fermentation. In the immobilized fermentation, 24.4 L/L_-broth_ gas was produced, which was 1.23 times than that of the free cells, this phenomenon indicated that TSH06 grew well in the immobilized system. However, the density of the suspended cells was obviously lower than that of free cell system, which suggested that most of the cells were adsorbed to the carriers. After 48 h, the cell growth entered the declining phase and the OD in free cell fermentation decreased gradually. In contrast, no obvious decline was observed in immobilized cell fermentation.

**Fig. 2:**
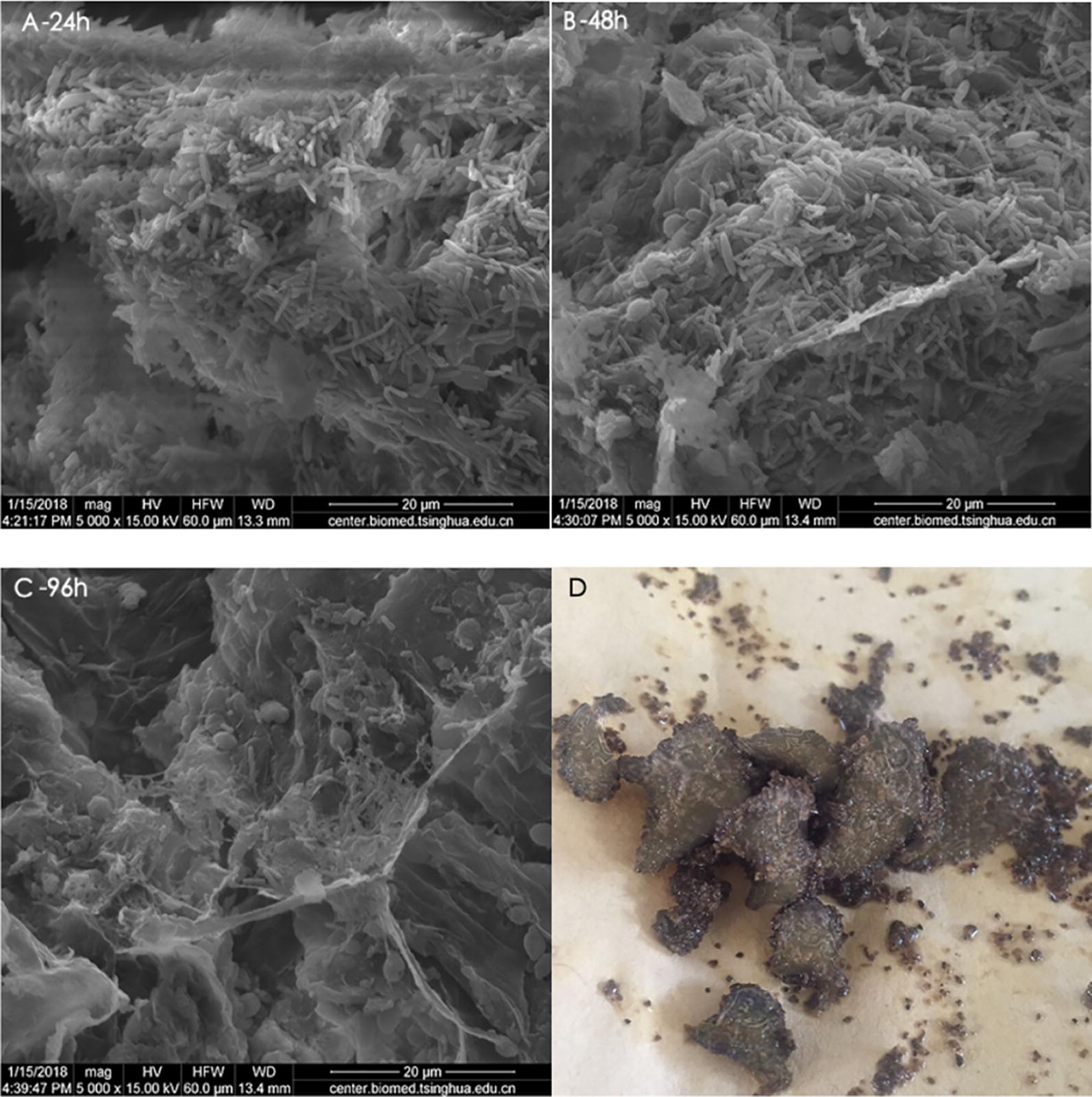
**A** 24 h: scanning electron microscope images of immobilized cell morphology at 24 h. **B** 48 h: scanning electron microscope images of immobilized cell morphology at 48 h. **C** 96 h: scanning electron microscope images of immobilized cell morphology at 96 h. **D** Cassava peel residues after cell immobilization

The surfaces of cassava peel residues before and after the cell immobilization were photographed by scanning electron microscope, and the pictures are shown in Fig. 2. The cells were immobilized onto cassava peel surfaces by adsorption. The cassava residue showed good cell adherence, this was probably because of its rough structure and the cells could attach and grow on its porous surface. The scanning electron microscope images of *C.acetobutylicum* (TSH1), *B.cereus* (TSH2) and TSH06 are shown in Additional file 1: Figure S3.

At 24 h of fermentation, many cells were adsorbed on the surface of the cassava peel and bar-shaped high-vitality bacteria existed as the dominant cell morphology. After culturing for 48 h, there was still a large amount of cells adsorbed on the immobilization carriers. At 96 h, the amount of cells adsorbed on the carriers significantly reduced and few spores were observed on the cassava peel surfaces, which indicated that spores and cell debris were barely able to be adsorbed on the cassava peel surfaces.

According to the results obtained from Figs. 1 and 2, the addition of cassava peel improved ABE production and a total solvents concentration of 20 g/L was achieved in the immobilized cell system with a productivity of 0.28 g/L/h, which showed the feasibility in ABE fermentation with cassava residue as an immobilized carrier.

### Continuous fermentation with free and immobilized cells

**Fig. 3:**
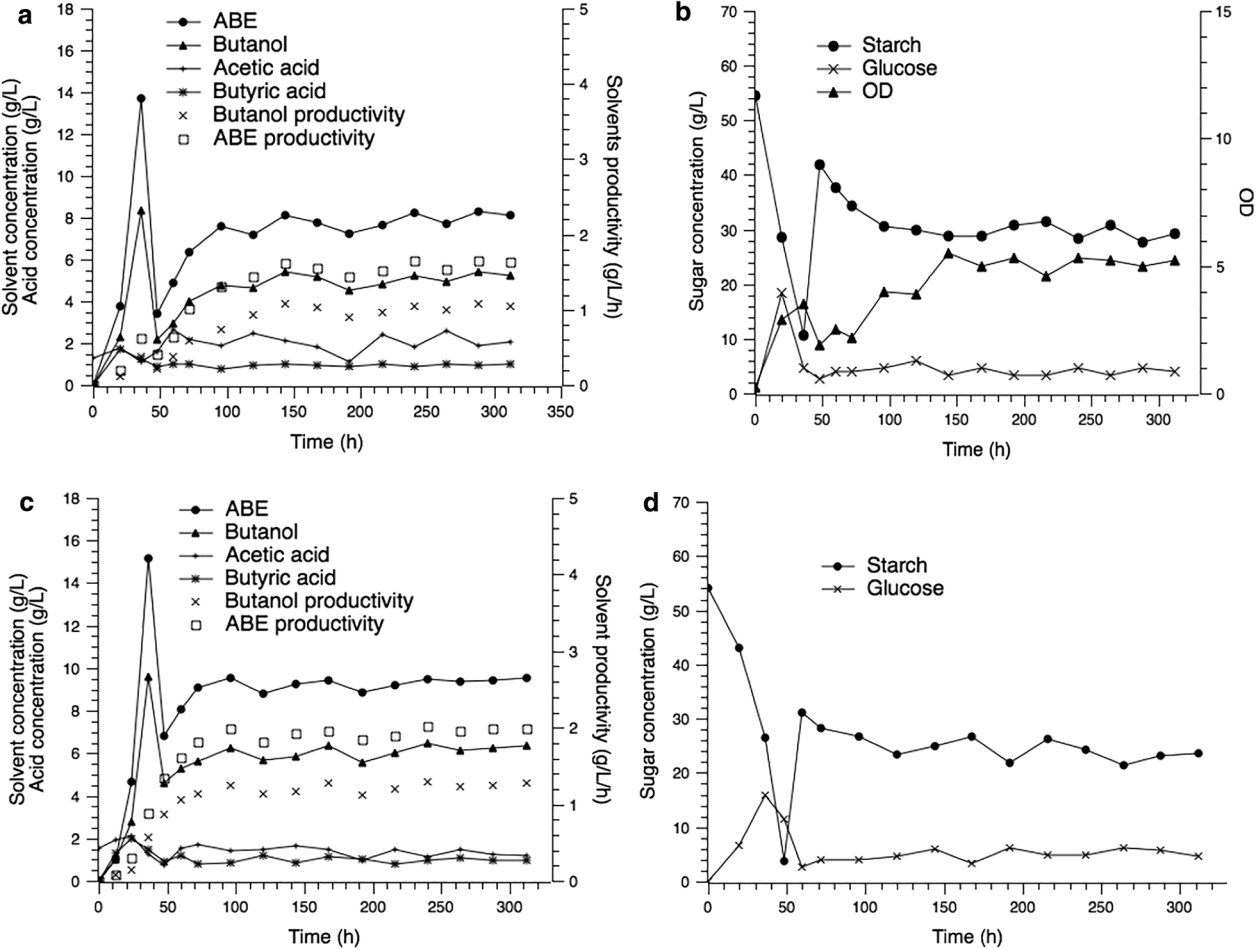
Continuous fermentation with free and immobilized cells. **a** Solvents production in the free cell system. **b** Substrate consumption and cell growth in the free cell system. **c** Solvents production in the immobilized cell system. **d** Substrate consumption and cell growth in the immobilized cell system

Compared with batch fermentation, continuous fermentation could effectively improve the solvent productivity and shorten the downtime [16]. In this study, continuous ABE fermentations with immobilized cells and free cells were compared. The first 36 h of fermentation represented the batch stage. Following this stage, the continuous fermentation was conducted by feeding 70 g/L of cassava solution at 100 mL/h. The dilution rate was 0.2/h (5 h of retention time). The kinetic curves of the continuous fermentations with free cells and immobilized cells are shown in Fig. 3.

For the free cell continuous fermentation, the chemostat culture was started when the butanol concentration reached 8.3 g/L. After 12 h of chemostat culture, the concentration of total ABE decreased to 5.5 g/L, which indicated that the strain was in the transition period from batch fermentation to continuous fermentation. The solvent concentrations increased gradually after 60 h and a relative constant concentration of butanol (between 4.7 and 5.3 g/L) was obtained after 96 h. The average butanol productivity and yield was 0.98 g/L/h and 0.24 g/g, respectively, and the residue starch concentration was 29.2 g/L.

For the immobilized cell continuous fermentation, the chemostat culture was started at 36 h. At that moment, the concentration of starch was 3.7 g/L and the butanol concentration was 9.6 g/L. The fermentation was quite stable after 96 h, and the total ABE concentration was 9.3 g/L, with 24.4% improvement compared to that of free cells. A butanol concentration of 6.3 g/L was observed with a residue starch concentration of 23.6 g/L. In the immobilized continuous fermentation, the butanol productivity was 1.26 g/L/h, which was 28.6% higher than that of free cell system. An ABE productivity of 1.86 g/L/h was achieved and the total ABE and butanol yields were 0.35 g/g_starch_ and 0.24 g/g_starch_, respectively.

No significant deterioration in ABE synthesis was observed after a total of over 300 h of continuous cultivation with immobilized cells. The results showed that the full utilization system of cassava was suitable for the long-term butanol production.

### Three-stage continuous fermentation with the full utilization of cassava

**Fig. 4:**
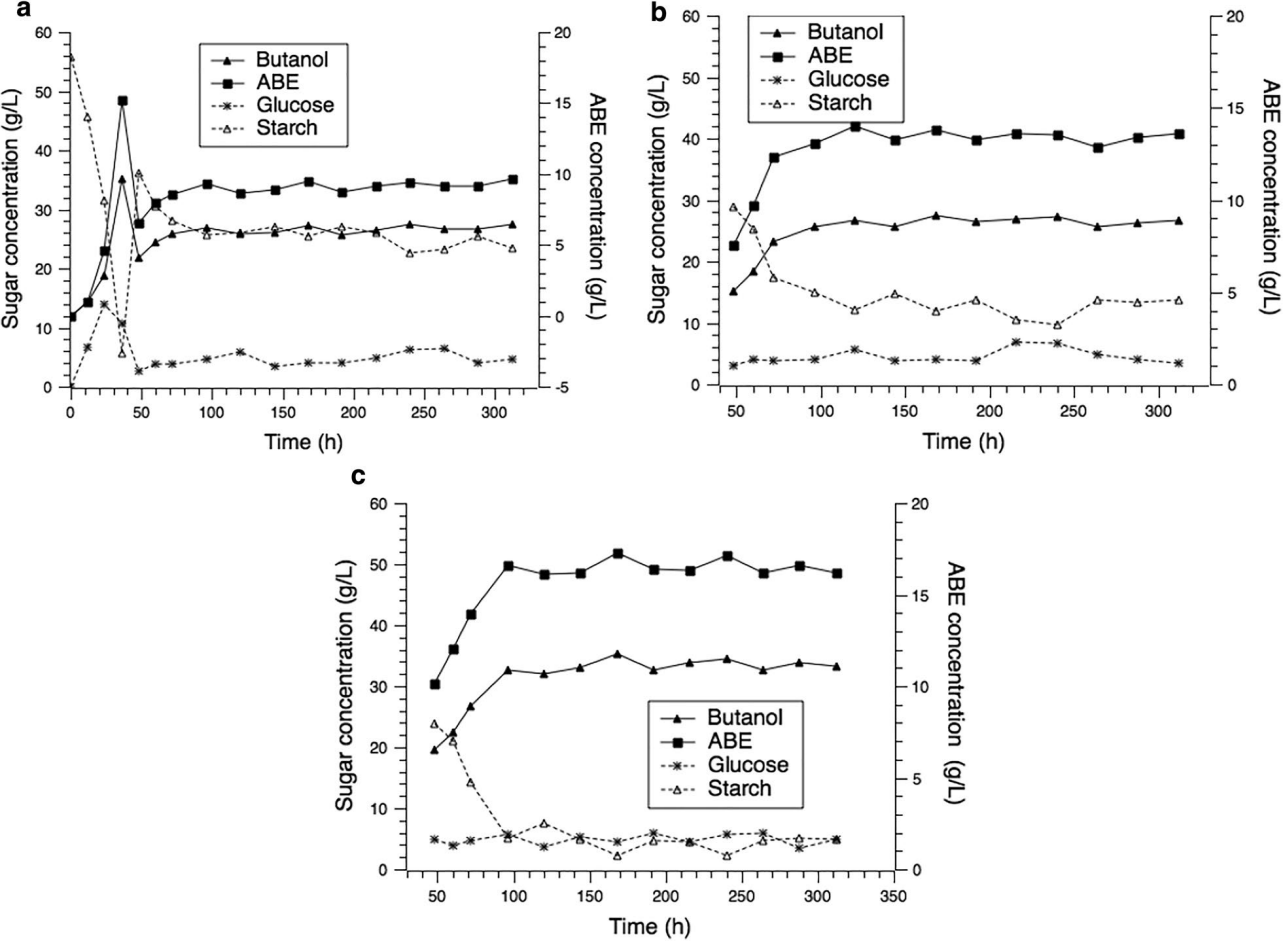
Three-stage continuous ABE fermentation with immobilized cell. **a** Solvents production and substrate consumption in the first stage. **b** Solvents production and substrate consumption in the second stage. **c** Solvents production and substrate consumption in the third stage

Three-stage continuous fermentation with cell immobilization was investigated to further improve the overall fermentation performance. The three-stage continuous fermentation was conducted in 1-L bioreactors with each stage maintaining 500 mL medium. The continuous fermentation was started at 36 h (the butanol concentration in the first reactor increased to 8 g/L). The feed medium was pumped to the first-stage reactor at a flow rate of 100 mL/h and the broth in each reactor was pumped to the next-stage reactor at the same flow rate to maintain constant reactor volumes. The broth in the third-stage reactor was pumped to a storage tank. The cassava flour was used as the substrate and cassava peel residues were used as immobilized carriers. The results are shown in Fig. 4.

A steady period was obtained in all three reactors after 96 h. The total solvent concentration in the first stage was 9.5 g/L. The starch concentration was maintained at 10.8–14.7 g/L. The solvent concentrations enhanced gradually with the increase of the reactor stage. The final ABE and butanol concentration reached 16.8 g/L and 11.2 g/L, respectively. At the same time, the starch concentration decreased to 2.3–5.2 g/L, which showed that the starch utilization rate increased from 48.5 to 85.8%. The butanol and total ABE productivity of the whole system were 0.75 g/L/h and 1.13 g/L/h, respectively. However, the butanol yield did not increase significantly and was still 0.24 g/g.

Table 2 shows the results of single-stage and three-stage continuous fermentations. In the three-stage continuous fermentation, the butanol concentration increased by 77.8% compared to the single-stage continuous fermentation. The final butanol and ABE concentration was 11.2 g/L and 16.8 g/L, respectively. However, butanol productivity decreased to 0.75 g/L/h, which was caused by the decrease of the dilution rate. In the three-stage continuous fermentation, the dilution rate in each reactor was set at 0.2/h, which corresponded to 0.067/h for the overall dilution rate. According to the description reported by Chang et al. and Survase et al., in a certain range of the dilution rate (approximately < 0.3/h), the ABE productivity increased with the increase of the dilution rate [8, 17]. Another reason is product inhibition, which has a negative effect on product formation rate. The butanol concentration in the first rector was 6.4 g/L, which was lower than the toxicity limit. The butanol concentrations in the second and third reactor were above 8.0 g/L, which could inhibit the cell growth and ABE synthesis [16]. Thus, the butanol concentration increased by 43.8% from the first-stage reactor to the second-stage reactor, while its concentration increased by only 21.7% from the second-stage reactor to the third-stage reactor.

**Table 2:** ABE production in single-stage and three-stage continuous fermentations

Parameters	Single-stage continuous fermentation	Three-stage continuous fermentation
Dilution rate (h^−1^)	0.2	0.2
Initial starch (g/L)	54.2	55.6
Residue starch (g/L)	23.7	3.6
Residue glucose (g/L)	4.7	4.8
Butanol (g/L)	6.3	11.2
ABE (g/L)	9.3	16.8
Butanol yield (g/g)	0.24	0.24
Butanol productivity (g/L/h)	1.26	0.75
ABE productivity (g/L/h)	1.86	1.12
Substrate utilization rate (%)	48.5	85.8

## Discussion

### ABE fermentation with non-pretreated cassava substrate

Cassava starch is a promising feedstock for ABE production due to its abundant availability, low cost and non-competition with direct food supplies. Some studies on butanol production from cassava have been conducted in recent years. Li et al. studied ABE fermentation with cassava starch hydrolysate as a substrate by *Clostridium* SE25, the butanol production was 16.2 g/L with a productivity of 0.23 g/L/h [18]. Similarly, Thang et al. reported butanol production with gelatinized cassava starch as a substrate by *Clostridium saccharoperbutylacetonicum* N1-4, a butanol production of 16.9 g/L was achieved with a butanol productivity of 0.23 g/L/h [3]. The ABE fermentation with cassava starch hydrolysate by *Clostridium beijerinckii* BA101 resulted in the highest butanol concentration reported so far, the butanol concentration was 25.7 g/L [5]. Other studies regarding ABE fermentation with cassava are presented in Table 3.

**Table 3:** ABE fermentations with cassava as the substrate

Organism	Fermentation mode	Pretreatment	Butanol (g/L)	Total solvents (g/L)	Butanol yield (g/g)	Butanol productivity (g/L/h)	Substrate utilization (g/L/h)	References
*C. acetobutylicum* SE25	Batch fermentation	Enzymatic hydrolysis	16.2	–	0.26	0.23	0.86	[18]
*C. butyricum* TISTR1032	Batch fermentation	Enzymatic hydrolysis	6.7	8.9	–	0.17	0.56	[13]
*C. beijerinckii* BA101	Batch fermentation	Enzymatic hydrolysis	25.7	37.0	–	0.31	–	[5]
*C. saccharoperbutylacetonicum* N1-4	Batch fermentation	Gelatinization	16.9	21.0	0.33	0.23	0.65	[3]
Mutant strain ART18 of *C. acetobutylicum* PW12	Batch fermentation	Gelatinization	15.8	23.2	0.31	0.19	0.59	[2]
*Clostridium* sp. strain BOH3	Batch fermentation	Gelatinization	17.8	24.2	0.3	0.25	0.82	[30]
Mutant strain SE36 of *C. acetobutylicum*	Batch fermentation	Gelatinization	16.1	24.9	0.32	0.20	–	[31]
TSH06	Batch fermentation	No pretreatment	13.3	20	0.24	0.18	0.75	This study
TSH06	Continuous fermentation	No pretreatment	11.2	16.8	0.24	0.75	3.13	This study

However, in previous studies, the fermentation substrates used in butanol production were mainly cassava starch hydrolysate or gelatinized cassava starch. In this study, fermentation with non-pretreated cassava flour was investigated by TSH06. In batch fermentation, the concentration of total ABE was 20 g/L with a productivity of 0.28 g/L/h, which was similar to that obtained with glucose as the substrate.

The symbiotic TSH06 was developed in our previous work, which could grow and produce butanol under non-strict anaerobic conditions [10, 11]. The results show that besides superior properties in oxygen tolerance, this strain has the ability to directly ferment starch, and thus, it is practical to utilize cassava with a simplified procedure, which is very important for economizing the process of biobutanol production.

### Immobilized cell fermentation with the full utilization of cassava

ABE fermentation coupled with cell immobilization technique was an effective approach to achieve high system stability and solvent productivity [19]. The methods of cell immobilization include adsorption, covalent bond formation and entrapment [20]. Particularly, adsorption is widely used because of its mild conditions, simple operation and no effect on cell growth [15]. In this study, ABE fermentation was conducted with cassava peel residue as an immobilized carrier. In the immobilized batch fermentation, butanol and total ABE production increased by 14.7% and 13.6%, respectively, compared to free cells. In the immobilized continuous fermentation, the butanol productivity was 1.26 g/L/h, with 28.6% improvement compared to the free cell system (0.98 g/L/h). This was mainly because the cells adhered to the porous surface, which was a microenvironment with small fermentable volume for cell metabolism [8]. Hence, the metabolism of the strain was promoted, which improved the consumption of substrate and the synthesis of product [9, 21].

Another possible reason was that biofilm forming on the surface of the carriers protected the inner cells by acting as barriers to chemical transfers [22]. The cell absorption on the cassava peel residue is shown in Fig. 2, and a lot of cells were adsorbed on the surface of the cassava peel, and bar-shaped high-vitality bacteria existed as the dominant cell morphology during the acidogenic phase (before 24 h) and the solventogenic phase (24–72 h). In addition, a milky biofilm was observed on the surface of the cassava peel residue. Sand et al. reported that biofilm is made up of microbial cells and extracellular polymeric substances and it has a complicated three-dimensional structure which provides an internal protective environment for microbial cells [21, 23, 24]. Extracellular polymeric substances contain extracellular proteins, polysaccharides (main support structure), nucleic acids and lipids, which could inhibit the diffusion of harmful substances helpful for the biofilm architecture maintaining inherent heterogeneity and complexity [22, 24]. A similar phenomenon was found by Zhuang et al., and the biofilm enhanced the tolerance of *C. acetobutylicum* to butanol. Supplementing butanol of 3 g/L at 24 h resulted in final butanol concentration of 4.8 g/L, while 8.6 g/L of butanol was produced by the immobilized cells [25].

The interspecies relationship between *C. acetobutylicum* and *B. cereus* were investigated in our previous study. *B. cereus* was found to deplete oxygen and provide anaerobic environment for *C. acetobutylicum* [11]. At the initial of fermentation, *B. cereus* grew first and exhausted the residual oxygen in fermenter, then *C. acetobutylicum* started to grow and produce solvents. Along with the fermentation, *C. acetobutylicum* was the dominant species and accounted for 99.85% of the whole population in solventogenic phase [11]. Therefore, it was probably that *C. acetobutylicum* was the dominant species which was adsorbed on the surfaces of the carriers.

Currently, agricultural residues, such as wood pulp, sweet sorghum bagasse and corn stalk bagasse, are commonly used as immobilization materials [8, 17, 21]. Chang et al. reported that when *C. acetobutylicum* was immobilized with corn stalk bagasse in a continuous ABE fermentation, 19.93 g/L of ABE was produced with a productivity of 0.8 g/L/h [21]. Chang et al. used sweet sorghum bagasse for cell immobilization, in the continuous fermentation, the ABE concentration and productivity was 16.5 g/L and 1.32 g/L/h, respectively [8].

### Multistage continuous fermentation

Batch fermentation is widely used in industry because it is easy to operate and low risk to be contaminated [26]. However, batch ABE fermentation has several drawbacks such as low productivity and low production due to product inhibition [27, 28]. Fed-batch mode by feeding concentrated medium cannot be applied with ABE fermentation without in situ removal of butanol to alleviate its severe inhibition to cells [16, 28]. Continuous fermentations have some advantages compared to batch processes: a significant reduction in downtime by elimination of idle times (charge, discharge, cleaning and sterilization of vessel), higher productivity and easier automatic operation [16]. Its high productivity and low lag-phase make it more suitable for large-scale butanol fermentation in comparison with batch mode [27]. However, the traditional one-stage free cell continuous fermentation may provide higher volumetric productivity but lower product concentration than batch fermentation [15]. One approach to solve the problem was to apply a continuous bioreactor with immobilized cells in the ABE fermentation process [15]. By the assistant of the immobilized carrier, the stability of the fermentation system could be promoted as well [8]. Another approach is to operate the fermentation in multistage mode [21]. In this way, the volumetric productivity and final product concentration of solvents showed a rising trend in a long term of fermentation [21, 29]. The three-stage fermentation process was investigated in this study. Compared with single-stage continuous fermentation, the concentration of butanol and total solvents increased by 77.8% and 55.6%, respectively. The final butanol and total solvent concentration reached 11.2 g/L and 16.8 g/L, respectively. In addition, the butanol and total solvent productivity was 0.75 g/L/h and 1.12 g/L/h, respectively. Both high solvent concentration and productivity were obtained in three-stage continuous fermentation.

Multistage fermentation has been reported for ABE production in some studies [15]. However, up to now, most studies focusing on multiple-stage continuous ABE fermentation were performed with glucose as the substrate. Chang et al. studied three-stage fermentation with corn stalk juice as the substrate, and ABE concentration and productivity was 19.9 g/L and 0.8 g/L/h, respectively [21]. However, in this work, a higher ABE productivity of 1.12 g/L/h was achieved, which was 40% higher than that obtained in that report.

## Conclusions

In this study, cost-effective ABE fermentation with full utilization of cassava by symbiotic TSH06 was shown. The results suggest that TSH06 can utilize cassava to efficiently produce ABE. In batch fermentation, an ABE concentration of 17.5 g/L was obtained with a productivity of 0.24 g/L/h, and these values were comparable to that of glucose as the substrate. Fermentation with immobilized cells was further studied and the cassava peel residue was used as immobilized carriers. In the immobilized system, the ABE productivity reached 0.28 g/L/h, with 16.7% improvement compared to that of free cells. The immobilized single-stage continuous fermentation resulted in a total ABE concentration of 9.3 g/L with a productivity of 1.86 g/L/h. In addition, both of the high solvent concentration and productivity were obtained in the three-stage continuous fermentation. The concentration and productivity of total ABE were 16.8 g/L and 1.12 g/L/h, respectively. These promising results provided feasibility for the industrial application of cassava butanol.

## Supplementary information

**Additional file 1: Figure S1.** Schematic diagram for three-stage continuous fermentation. **Figure S2.** Starch testing of cassava peel with iodine solution. **Figure S3.** Scanning electron microscope images of *C. acetobutylicum* (TSH1), *B. cereus* (TSH2) and TSH06.

## Data Availability

All data generated or analyzed during this study are included in this published article and its additional files.

## Abbreviation

ABE: acetone–butanol–ethanol
