# Supplementary material for: Effective continuous acetone–butanol–ethanol production with full utilization of cassava by immobilized symbiotic TSH06
Source: Biotechnol Biofuels. 2019 Sep 16;12:219. doi: 10.1186/s13068-019-1561-1 (PMC6745785; doi:10.1186/s13068-019-1561-1)
Supplement: Supplementary file 1 — Additional file 1: Figure S1. Schematic diagram for three-stage continuous fermentation. Figure S2. Starch testing of cassava peel with iodine solution. Figure S3. Scanning electron microscope images of C. acetobutylicum (TSH1), B. cereus (TSH2) and TSH06. [file 13068_2019_1561_MOESM1_ESM.docx]

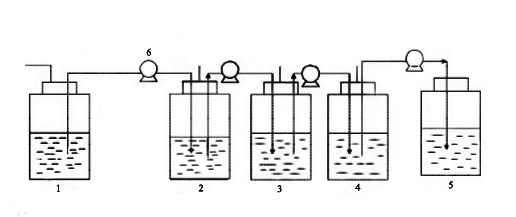


Figure S1 Schematic diagram for three-stage continuous fermentation

(1. Substrate tank 2. Fermenter 3. Fermenter 4. Fermenter 5. Storage tank 6. Peristaltic pump)


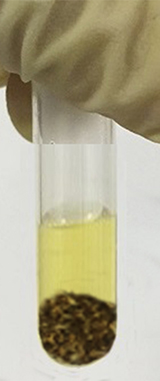


Figure S2 Starch testing of cassava peel with iodine solution


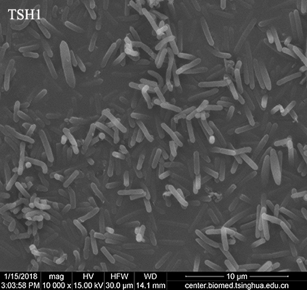

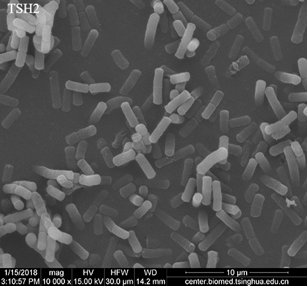

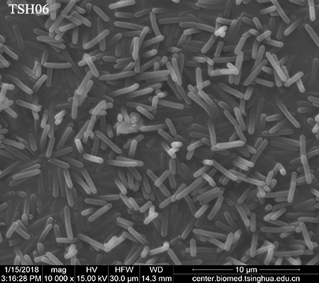


Figure S3 Scanning electron microscope images of *C. acetobutylicum* (TSH1) , *B. cereus* (TSH2) and TSH06
